# “Your Tumour Can Save Lives”: Re-examining Singapore’s Consent Procedures for the Use of De-identified Bio-specimens in Research

**DOI:** 10.1007/s41649-024-00327-z

**Published:** 2025-04-30

**Authors:** Kylie J. Q. Heng, Shaun S. E. Loong, Gini W. W. Wong, Athena Ham, Aaron D’Sa, Mayank Dalakoti, Roger Foo, Jerry Menikoff

**Affiliations:** 1 Cardiovascular Metabolic Translational Research Programme, Yong Loo Lin School of Medicine, National University of Singapore, Singapore; 2 Duke-NUS Medical School, Singapore; 3 University of Cambridge, Cambridge, England UK; 4 Department of Cardiology, National University Heart Centre, National University Health System, Singapore; 5 Faculty of Law, National University of Singapore, Singapore; 6 Centre for Biomedical Ethics, Yong Loo Lin School of Medicine, National University of Singapore, Singapore; 7 British Heart Foundation Cardiovascular Epidemiology Unit, Department of Public Health and Primary Care, University of Cambridge, Cambridge, UK

**Keywords:** Research ethics, Consent, De-identified bio-specimens, Law, Human biomedical research, Biomedical ethics

## Abstract

Consent in research is inarguably a highly contentious and debated topic. One of the biggest debates in research ethics revolves around the determination of when consent is necessary, as there is a need to balance participant protections and research advancements. Contrary to popular belief, obtaining informed consent may not necessarily be better for the participant. One illustration of this can be taken from the United States’ (US) 2011 proposal to require consent for the research use of de-identified bio-specimens under the applicable regulations (what is often referred to as the “Common Rule”). The proposed transition from no consent to broad consent was met with strong opposition from researchers and the public alike, highlighting the possible superiority of not requiring consent when de-identified bio-specimens are used in research. Against this backdrop, this article uses legal and bioethical perspectives to evaluate Singapore’s consent procedures for the secondary research use of de-identified bio-specimens, arguing that Singapore is ready and in need of further liberties to allow for more robust research advancements and better participant outcomes.

## Introduction

Consent in research is inarguably a highly contentious and debated topic, as the standard for appropriate consent-taking is continually changing with shifts in societal attitudes and the emergence of new technological advancements. Whilst the core purpose of consent—respecting participants’ autonomy and ensuring their welfare and protection (Gupta 2013)—remains unchanged, there is continual debate on how to best structure consent procedures, including determining when consent is even necessary, to strike a balance between participant protection and research advancement.

Contrary to popular belief, obtaining informed consent may not always be better for participants. When informed consent is inappropriately mandated, the advancement of important life-saving medical research is unnecessarily impeded. Further, as we will expound below, it may even prove to be harmful by creating additional barriers to minority research participation, potentially exacerbating inequalities in research representation and, paradoxically, increasing privacy risks by serving as an unnecessary link to participants.

One illustration of where informed consent was recognised to be inappropriately mandated can be taken from the United States’ (US) 2011 proposal to require consent for the research use of de-identified bio-specimens under the applicable regulations (what is often referred to as the “Common Rule”). The proposal would have required researchers to obtain written study-specific or broad consent before de-identified bio-specimens could be used in research. This would have been a dramatic change from the existing rules which do not mandate such consent (Kaiser 2017). Thus, the proposed transition from no consent to broad consent was met with strong opposition from researchers and the public alike, highlighting the possible superiority of not requiring consent when de-identified bio-specimens are used in research (Spector-Bagdady et al. 2019).

Against this backdrop, perhaps it is time for Singapore to re-examine its consent procedures for the secondary research use of de-identified bio-specimens. Perhaps further liberties should be applied to the consent-taking procedures to allow for more robust research advancements and better participant outcomes.

## What are De-identified Bio-specimens?

Bio-specimens are broadly defined, under US regulations, as any material derived biologically, including tissues or blood (U.S. Department of Health and Human Services n.d.). In alignment with the Human Biomedical Research Act 2015 (2020 Rev Ed) (the “HBRA”) which governs all medical research conducted with bio-specimens in Singapore, the definition of bio-specimens in this article does not include naturally excreted tissue (e.g. faeces and urine) with the exception of placental tissue (MOH 2021). These bio-specimens can be donated for research-specific use or derived as surplus from clinical procedures. For the purpose of this article, we will be focusing on the consent requirements for the secondary research use of de-identified bio-specimens derived as surplus from clinical procedures.

To be considered de-identified, bio-specimens must be stripped of direct identifiers and unable to be linked back to the donating individual by personnel involved in the project or accessing the bio-specimen (Goldenberg et al. 2015). In Singapore, the definition of de-identified bio-specimens includes both reversibly and irreversibly de-identified bio-specimens (Personal Data Protection Commission Singapore 2022). Irreversibly de-identified bio-specimens refer to permanently pseudonymised bio-specimens whose original identifiers are properly destroyed (Bernasconi et al. 2020) whereas reversibly de-identified bio-specimens refer to bio-specimens which retain linkages between the direct identifiers and pseudonyms.

## What is being Done in Singapore

In Singapore, the HBRA is the statute regulating human biomedical research. Generally, “appropriate consent” as defined under Part 3 of the HBRA must be obtained for the use of human tissues in research.^1^ Thus, when seeking consent to obtain human tissues, researchers must inform participants (or the relevant persons authorised to give consent on the participant’s behalf)^2^ of (a) the specific research purpose for which the tissue is intended to be used if this information is available or (b) the general purpose for which the tissue is intended to be used if the specific study purpose is unavailable.^3^ The aforementioned criteria appear to apply to both the primary and secondary research use of tissues, as the HBRA does not establish separate consent regimes for them. This lack of distinction sets the expectation that researchers should subject post-procedural surplus tissues and primary tissues collected for research to the same consent standards.

In short, this means that, from a general legal perspective, broad consent is the minimum required standard of consent-taking for the use of de-identified bio-specimens in research. Defined as consent for participation in future unspecified research studies (Grady et al. 2015), broad consent allows researchers greater flexibility (Maloy and Bass 2020) in consent-taking as they are not required to gain study-specific consent each time a bio-specimen is used for either primary or secondary research purposes.

## An Examination of Singapore’s Laws: Why there Needs to be a Change

Given that in practice broad consent appears to be an ethically permissible standard of consent-taking in Singapore, the positioning of broad consent as an alternative option to study-specific consent in the legislation appears to be counterintuitive. Whilst some clinicians have capitalised on the benefits of broad consent and routinely seek such consent for the secondary use of surplus tissues, this may not be uniformly practised.

Rather than a standardised recommended consent-taking procedure for the collection of surplus tissues, clinicians in Singapore are given the discretion to take consent according to their own interpretation of the law. In separate statements guiding researchers on the most appropriate consent-taking procedures for the collection and secondary use of surplus bio-specimens in research, both the Singapore Ministry of Health (MOH 2021) and SingHealth (2021) merely redirected researchers to the HBRA. By placing the onus of consent-taking on individual researchers, who may differ greatly in their willingness to collect and take consent for the secondary use of surplus tissues in every procedure, an unnecessary barrier is created in the collection of bio-specimens. In contrast, implementing a unified and standardised consent protocol would enhance the productivity of medical research and education, as the collection of surplus bio-specimens would be optimised (Zenker et al. 2022).

More importantly, the HBRA does not recognise that standard consent procedures for the use of de-identified bio-specimens should be made distinct from that of identifiable bio-specimens. In contrast to bio-specimens, there exists such a distinction for identifiable and de-identified health information. According to s25 HBRA, “appropriate consent” is required for the research use of “biological material or individually-identifiable health information”.^1^ Since the HBRA expressly distinguishes the consent requirement for identifiable and de-identified health information, we argue that the HBRA can also make such a distinction for identifiable and de-identified bio-specimens.

By referring to de-identified and identifiable biological material as a monolithic entity, in contrast to the specification of distinct rules for identifiable and de-identified health information, the legislation sets a precedent that bio-specimen use in research should be assessed with equal weighting regardless of its identifiability. This is disadvantageous to Singapore as, in contrast, other nations such as the US, United Kingdom (UK) and European Union (EU) have recognised the distinction in consent requirements given that the use of de-identified bio-specimens in research is generally regarded to have minimal risk (Goldenberg et al. 2015).

For example, the governing legislation in the UK, the Human Tissue Act 2004 (the “HT Act”) distinguishes the consent requirement for the removal, storage and use of human tissues as separate activities. This is in contrast to Singapore’s HBRA consent requirement covering both the removal and processing of tissues. There are a number of exceptions to the requirement of consent under the HT Act, such as where consent is not required for the storage and use of relevant material for the purposes of research if (i) the material was taken from the body of a living person, (ii) the material is non-identifiable and (iii) the research is ethically approved by an NHS research ethics authority.^2^ Thus, it is evident that the UK has separate consent criteria for identifiable and de-identified bio-specimens, even exempting the need for consent in the research use of de-identified bio-specimens.

Moreover, whilst there is no unified law specifically governing the use of de-identified bio-specimens in the EU, there is a dominant “consent or anonymise” approach in most member states (Sethi and Laurie 2013). This approach recognises anonymisation of genomic data and its associated bio-specimens as an adequate alternative to consent taking.

In view of this, Singapore should reform its consent procedure to promote the secondary research use of de-identified bio-specimens and maintain its competitive edge on an international scale.

## Lessons Learnt: Why Consent Exemption is a Superior Consent-Taking Model

In line with the practice of “anonymise or consent”, we argue that requiring consent for the secondary research use of de-identified bio-specimens is counterproductive and far too restrictive.

A requirement for consent not only increases the administrative burden on researchers and physicians (Association of American Universities 2011) but also poses a threat to medical advancements and patient care as it unnecessarily reduces the availability of bio-specimens. This is especially unfair for racial minorities and patients with rare diseases as the availability of bio-specimens is already limited by virtue of their small number size and is further worsened by this added barrier to the collection of their bio-specimens (Kaiser 2016). Being a predominantly Asian nation, there is an even greater pressing need for Singapore to reduce the barriers to medical research locally, as Asians are repeatedly underrepresented in global medical research. Whilst Asians make up approximately 60% of the total global population, a 2020 survey of global clinical trials found that Asians only represented 11% of the total participant cohort across clinical trials (Sharma and Palaniappan 2021) .

We acknowledge the fact that the use of de-identified bio-specimens in research has potential harms, such as the risk of re-identification of specimens or other possible breaches in privacy (Rothstein 2010). However, we disagree that consent-taking is the appropriate method of circumventing these risks.

The consent-taking procedure is a disproportionate barrier to research as compared to the potential benefits that society could reap from medical advancements using de-identified bio-specimens. For example, the identification of HER-2 which laid the foundation for immunotherapies against breast cancer was only possible due to the use of archival human tissues (Bledsoe and Grizzle 2013). Had there continued to be significant barriers to the collection of bio-specimens, HER-2-positive breast cancer would have continued to have a poor prognosis, resulting in the now-preventable deaths of thousands of individuals. In parallel to the consumer privacy paradox, where consumers are willing to relinquish some control over their privacy due to the increased perceived benefits of products, such as social media (Ho et al. 2023), the public will likely be willing to bear some risk of privacy breaches in exchange for these medical innovations.

This is not an entirely novel concept in the field of research ethics either, as the principle of proportionality has been commonly used to discuss the ethics of research methods (Hermerén, 2011). For example, this is recognised in the EU’s promulgation of articles 9(4) and 9(2)(j) in the General Data Protection Regulation which allows genetic data and its associated bio-specimens (Peloquin et al. 2020) to be processed for scientific research without consent, provided that the principle of proportionality and subjects’ protections are maintained (Staunton et al. 2019).

Further, having a consent-taking procedure does not actually directly minimise the risks of re-identification or other privacy breaches. Paradoxically, it actually increases the risks to participants. It makes it a requirement for de-identified bio-specimens to be tied to the consent forms for verification purposes, creating an unnecessary linkage between the bio-specimen and the donor, thus making re-identification easier (Arvin and Greenberg 2015) .

Hence, consent exemption should be considered as the consent standard for all secondary research studies involving de-identified bio-specimens in Singapore as consent-taking does not demonstrably improve participant welfare and unnecessarily slows down the progression of important medical research (Association of American Universities 2016).

From a standpoint of moral relativism, one might cite cultural differences and argue that consent is still required due to Singapore’s conservatism and traditional beliefs about the sanctity of human tissue which should be respected (Muthiah et al. 2021). However, this argument is contradicted by the presence of section 5.37 in the Bioethics Advisory Committee’s (BAC) Ethics Guidelines for Human Biomedical Research which allows IRBs to waive the consent requirement for the secondary use of surplus de-identified bio-specimens in research, if initial consent was unable to be obtained (Bioethics Advisory Committee Singapore 2021). The provision of a waver implicitly shows that the secondary research use of de-identified bio-specimens without consent is considered ethically permissible within Singaporean society.

Given that the cultural ethical considerations informing both the BAC’s guidelines and formal legislation are the same, it begs the question of why the Singapore legislature has yet to formally endorse the secondary research use of de-identified bio-specimens without consent. The key reason lies in the BAC’s provision that the consent waiver must be approved and deemed ethically permissible by the Institutional Review Board (IRB). Rather than a unified legislation allowing for the secondary research use of de-identified bio-specimens without consent, there is an insistence that ethics committees and IRBs need to review individual cases to discern whether the consent waiver is appropriate.

This is an unnecessary obstacle to the advancement of medical research. When ethics committees and IRBs are unnecessarily involved in the assessment of otherwise ethically unambiguous decisions, such as when bio-specimens are considered to be de-identified, research progress is needlessly stalled (Savulescu 2014). Instead of giving discretionary power to IRBs, which have been found to vary in their approved research practices (Silverman et al. 2001), focus should be placed on providing institutions and researchers with a clear-cut definition of when a bio-specimen is considered sufficiently de-identified and can hence be used without consent in secondary research.

## Will the Singapore Public be in Favour of a Formal Removal of Consent-Taking Procedures?

There appears to be a positive global trend of public support for the removal of active consent procedures (Eikemo et al. 2022) as the public becomes increasingly aware of the disproportionate relationship between active consent-taking procedures and the immense benefits of research using de-identified bio-specimens (Council on Governmental Relations 2016). However, the question remains as to whether Singapore, in particular, would be ready for a liberalisation of its consent-taking procedures. Whilst specific studies need to be done to understand the general public’s opinions on the implementation of a no-consent procedure for the secondary use of de-identified bio-specimens in research, the current literature suggests that Singaporeans are likely to be open to a more liberal consent-taking approach.

A study conducted on Singaporeans’ attitudes towards the sharing of their de-identified health information without re-consent found that a majority (67%) was willing to do so (Lysaght et al. 2021). They were even willing to share their genomic sequencing data as long as they could be assured of the legitimacy of its uses and of the de-identification process (Lysaght et al. 2020). In the context of bio-specimen collection in particular, it was found that 73.58% of Singaporeans undergoing procedures in NUH from 2002 to 2011 agreed to the donation of their residual tissues for research purposes (Chan 2014). Although it is acknowledged that this data pre-dates the HBRA which may have altered the way that public health institutions obtain consent for the secondary research use of surplus post-procedural bio-specimens, this data still hints at Singaporeans’ likely receptivity and openness to the secondary use of their de-identified bio-specimens even with reduced consent-taking procedures. This is especially so as studies have shown that consent-taking is not typically Singaporeans’ highest concern when it comes to the collection of their de-identified bio-specimens or health information for research (Lysaght et al. 2021). Rather, they are more concerned with the potential uses and with which users would have access to their bio-specimens and health data.

Whilst it is apparent that Singapore is ready and in need of a liberalisation in the consent procedures surrounding de-identified bio-specimens, we do acknowledge that it might take time for the public to agree on the complete removal of consent-taking. Hence, a revised consent regime need not necessarily be an implementation of the US’s no-consent procedure. Given Singapore’s culturally diverse population with varying religious and cultural beliefs, passive consent alternatives can be considered instead. One example in particular is an opt-out scheme where all individuals undergoing medical procedures are assumed to consent to the collection and de-identification of their surplus tissues, unless otherwise stated. This softer approach might be more palatable to the Singaporean public, especially given the precedence of their receptivity towards existing opt-out schemes in other contexts, such as organ donation (MOH, n.d.).

## Conclusion

Singapore’s current legislation on the consent procedures for the secondary research use of de-identified bio-specimens is counterproductive to medical research advancements. The requirement for consent is not only an unnecessary barrier to research that disproportionately harms minority groups but also perversely increases the privacy risks to research participants. It is, hence, time for us to re-examine the consent procedures in favour of greater liberties to pave the way for more medical research advancements which will directly benefit the local population and increase the quality of healthcare. Consent-taking may be an inadequate and unnecessary form of participant protection in the context of research involving de-identified bio-specimens. Instead, participant protection should be ensured through stronger de-identification processes and the formation of watchdogs to govern the use of de-identified bio-specimens in research.

Whilst there appears to be a promising pool of literature hinting at Singaporeans’ openness towards the liberalisation of consent-taking procedures for the secondary research use of de-identified bio-specimens, we acknowledge that more work needs to be done to truly understand the public’s opinion towards it. Future research should also be focussed on the public’s opinions on specific proposed changes in the law, such as the implementation of an opt-out approach for research with de-identified bio-specimens.

## Footnotes

1 HBRA s 25.

2 HT Act, s 1(7)–(9).
